# Longitudinal economic burden of incident complications among metabolic syndrome populations

**DOI:** 10.1186/s12933-024-02335-7

**Published:** 2024-07-10

**Authors:** Kah Suan Chong, Yi-Hsin Chang, Chun-Ting Yang, Chu-Kuang Chou, Huang‑Tz Ou, Shihchen Kuo

**Affiliations:** 1 Institute of Clinical Pharmacy and Pharmaceutical Sciences, College of Medicine, National Cheng Kung University, Tainan, Taiwan; 2 Department of Pharmacy, College of Medicine, National Cheng Kung University, Tainan, Taiwan; 3 Division of Pharmacoepidemiology and Pharmacoeconomics, Department of Medicine, Brigham and Women’s Hospital, Harvard Medical School, Boston, MA USA; 4 Division of Gastroenterology and Hepatology, Department of Internal Medicine, Ditmanson Medical Foundation Chia-Yi Christian Hospital, Chiayi, Taiwan; 5 Obesity Center, Ditmanson Medical Foundation Chia-Yi Christian Hospital, Chiayi, Taiwan; 6 Division of Metabolism, Endocrinology & Diabetes, Department of Internal Medicine, University of Michigan Medical School, Ann Arbor, MI USA

**Keywords:** Metabolic syndrome, Cardiovascular disease, Complications, Microvascular disease, Cancer, Cost

## Abstract

**Background:**

This study quantifies the longitudinal economic burden for a wide spectrum of incident complications, metabolic syndrome (MS)-related risk factors, and comorbidities in patients with MS.

**Methods:**

This retrospective study utilized linked data from the 2013 National Health Interview Survey and the 2012–2021 National Health Insurance Research Database to identify MS individuals and their characteristics. The incidence rate of each complication was calculated as the number of complication events in the study period divided by the total person-years during follow-up. The healthcare costs of complications were analyzed using a generalized estimating equation model to determine the cost impact of complications after adjustment for patients’ characteristics. Sensitivity analyses on variables with high missing rates (i.e., cause of death, body mass index) were performed.

**Results:**

Among 837 identified MS individuals over 8.28 (± 1.35) years of follow-up, the most frequent complications were microvascular diseases (incidence rate for nephropathy/retinopathy/neuropathy: 6.49/2.64/2.08 events per 100 person-years), followed by cardiovascular diseases (2.47), peripheral vascular diseases (2.01), and cancers (1.53). Death was the costliest event (event-year cost per person: USD 16,429) and cancers were the most expensive complications (USD 9,127−11,083 for non-MS- and MS-related cancers). Developing non-MS/MS-related cancers, cardiovascular diseases, and obesity-related medical conditions increased annual costs by 273% (95% CI: 181−397%)/175% (105−269%), 159% (118−207%), and 140% (84−214%), respectively. Microvascular diseases had the lowest cost impact on annual costs (i.e., 27% [17−39%]/27% [11−46%]/24% [11−37%] increases for nephropathy/neuropathy/retinopathy, respectively). Having existing comorbidities increased annual costs by 20% (osteoarthritis) to 108% (depression). Having morbid obesity (i.e., body mass index ≥ 35 kg/m^2^) increased annual costs by 58% (30−91%).

**Conclusions:**

The economic burden from costly incident complications (i.e., cardiovascular diseases, peripheral vascular diseases, cancers), MS-related risk factors (i.e., morbid obesity), and comorbidities (i.e., depression) highlight the urgent need for early intervention to prevent MS and its progression. The comprehensive cost estimates reported in this study can facilitate the parameterization of economic analyses to identify cost-effective interventions for these patients.

**Supplementary Information:**

The online version contains supplementary material available at 10.1186/s12933-024-02335-7.

## Introduction

Metabolic syndrome (MS) comprises a large array of cardiometabolic risk factors, which typically include central obesity, insulin resistance, hypertension, and dyslipidemia, that greatly increase the risk of developing numerous chronic disorders (e.g., cardiovascular diseases [CVDs], diabetes, chronic kidney diseases, and cancers) [1]. A rising prevalence of MS has been observed in Asian (from 19.9% in 2011–2012 to 26.2% in 2015–2016) [2] and Taiwan populations (from 13.6% in 1993–1996 to 25.5% in 2005–2008) [3]. MS has a considerable economic impact, highlighting the urgent need for early initiatives and preventive programs [4, 5]. Overall, MS increases health utilization and medical expenditures, with an estimated 1.2- to 2.2-fold increase compared with non-MS [6, 7]. This burden can be amplified by the presence of MS-related risk factors and the development of associated complications. On average, the total annual healthcare cost of MS cases increased by 24% for an additional risk factor related to MS [7]. The cumulative costs of having new onset diabetes and CVDs in MS populations in a 10-year simulation were estimated at USD 7,700 and USD 1,614−2,731 per case, respectively [8].

However, most studies have mainly focused on the costs of MS-related risk factors, including abnormal blood pressure, lipid profile, blood glucose level, and high waist circumference [6, 7, 9, 10]. A few studies investigated some complications (e.g., CVDs) associated with MS [6, 8, 10]. Among these studies, the simulation analyses [8] or studies with limited follow-up periods (e.g., 5 years [6, 10]) that targeted certain complications might not reflect the disease progression of real-world MS populations. Moreover, given the high economic impact of comorbidities, including depression and osteoarthritis, in individuals with interrelated conditions (i.e., obesity [11], diabetes [12]) with MS, the extent to which these comorbidities impact MS populations should be investigated. Unfortunately, there is a lack of evidence on the economic burden attributable to the development of various complications, including microvascular events, cancers, metabolic complications, and obesity-related disorders (e.g., sleep apnea, a clinically important disorder in individuals at risk of obesity), MS-related risk factors, and comorbidities in MS populations. Such evidence is critical for parameterizing the economic burden of MS-related clinical events and facilitating the development of timely interventions and efficient allocation of healthcare resources for the early prevention or alleviation of costly drivers in these populations. In addition, considering the substantial economic burden caused by the rising trend of MS and numerous chronic health problems during its progression [2, 3, 13], longitudinal data are warranted to quantify the long-term health and economic consequences for MS individuals to provide an overview of the MS-associated burden to individuals and society.

Against this background, this nationwide population-based study seeks to determine the economic burden of a wide spectrum of incident complications, MS-related risk factors, and comorbidities in patients with MS over 9 years of follow-up.

## Methods

### Data sources

This retrospective study utilized two nationwide datasets, namely the National Health Interview Survey (NHIS) [14] for 2013 and the National Health Insurance Research Database (NHIRD) [15] for the period from 2012 to 2021. These two datasets were linked using individual, encrypted, de-identified numbers by the Health and Welfare Data Science Center (detailed description in Supplementary Method 1). This study was approved by the Institutional Review Board of National Cheng Kung University Hospital (A-EX-109-035).

### Definition of study population

In this study, the presence of MS was defined as having any three or more of the following MS-related risk factors: body mass index (BMI) ≥ 27 kg/m^2^ [16], hypertension, diabetes, and hyperlipidemia (as identified from the linked NHIS-NHIRD for 2013). An individual’s BMI level was estimated using the height and weight records in the NHIS. The presence of hypertension, diabetes, and hyperlipidemia was defined as having disease diagnoses and/or exposure to associated treatments in the NHIRD (Supplementary Table 1). The date of MS confirmation (i.e., the date when patients met the study criteria of MS) was defined as the index date. Patients < 18 years old at the index date or with missing weight, height, or personal identification number data were excluded. Of note, patients with any prior MS-related complications (i.e., macrovascular/microvascular/metabolic complications, obesity-related medical conditions, and cancers) in the year before the index date were also excluded (Supplementary Table 1).

### Identification of baseline patient characteristics, comorbidities, complications, and costs

Patient characteristics, including demographics (i.e., age, sex), BMI, smoking history, betel nut history, education level, marital status, and household income level at the index date, were identified from the NHIS. The presence of MS-related risk factors (e.g., hypertension, diabetes, hyperlipidemia) and comorbidities (i.e., osteoarthritis, and depression) was ascertained in the year before and at the index date based on the NHIRD.

Following previous studies [12, 17–21], several MS-related complications of interest were measured from the index date to the end of the study follow-up (i.e., death or December 31, 2021, whichever came first), including: (1) macrovascular events (i.e., CVDs, cerebrovascular diseases, and peripheral vascular diseases), (2) microvascular events (i.e., nephropathy, retinopathy, and neuropathy), (3) metabolic events, (4) other obesity-related medical conditions (i.e., sleep apnea, bariatric surgery, and knee replacement therapy), and (5) cancers (i.e., MS-related cancers such as liver cancers and non-MS-related cancers [21]). The above comorbidities and complications were measured using the International Classification of Diseases, 9th and 10th Revisions, Clinical Modification (ICD-9-CM and ICD-10-CM) disease diagnosis codes (Supplementary Table 1) and the obesity-related medical conditions were mainly identified using the International Classification of Diseases, 9th and 10th Revisions, Procedure Coding System (ICD-9-PCS and ICD-10-PCS) in the outpatient and inpatient department files of the NHIRD.

The economic analysis in this study was conducted from the perspective of the healthcare sector, so we only measured the direct medical costs attributable to outpatient visits, hospital admissions, emergency room visits, and prescriptions, which were reimbursed by the National Health Insurance program and co-paid from patients, from the year before the index date through the end of the study follow-up. In particular, the crude healthcare costs of MS-related complications of interest were estimated in terms of event-year and annual state-year costs (Supplementary Method 2). All costs were converted to year 2023 values using the medical component of the consumer price index in Taiwan and are presented in United States dollars (USD).

### Statistical analyses

Descriptive analyses were performed. The means and standard deviations (SDs) are presented for continuous variables and percentages are presented for dichotomized/categorical variables. Differences in the descriptive statistics of baseline characteristics between gender groups were examined using the *t*-test, Fisher’s exact test, and the chi-square test, whichever was most appropriate. The incidence rate ratio test was performed to examine differences in the incidence rate between age (i.e., ≥ 65 years versus < 65 years) and gender groups. A *p*-value of less than 0.05 was considered to indicate a statistically significant difference. The incidence rate of each complication was calculated as the number of complications of interest in the study period divided by the total observation time of individual patients. The incidence rate for each complication is presented as the number of events per 100 person-years. The cumulative incidence of each complication was measured from the index date to the end of follow-up using the life table method (SAS LIFETEST procedure).

The generalized estimating equation (GEE) model with a log-link function was adopted to assess the economic impact of MS-related complications over the study follow-up period with adjustment for patients’ baseline characteristics and comorbidities. Given the log-transformed cost data, the coefficients and associated 95% confidence intervals (CIs) derived from the GEE model were back-transformed to the ordinal scale using an exponential function for cost multipliers (Supplementary Method 3).

We conducted a series of sensitivity analyses to ensure the robustness of the study results (Supplementary Tables 2−6). First, deaths from specific causes (i.e., fatal CVDs, fatal MS-related cancers) and other causes of death were specified in the GEE analysis. Second, considering the discrepancy in the BMI cut-off point for obesity between Asian (i.e., 27 kg/m^2^) and Western settings (i.e., 30 kg/m^2^), different cut-off points were considered (i.e., 27 and 30 kg/m^2^ considered as obesity in different settings [22–24] and 35 kg/m^2^ considered as morbid obesity) in the sensitivity analyses. Third, given considerable unknown data on smoking status (> 50%), this variable was excluded from the analysis. We also performed a GEE analysis using the study variables without any unknown data (i.e., the variables of smoking, education level, and monthly household income were excluded). All statistical analyses were performed using SAS software version 9.4 (SAS Institute, Cary, NC). The Strengthening the Reporting of Observational Studies in Epidemiology (STROBE) checklist is provided in Supplementary Table 7.

## Results

A total of 837 individuals (53.88% male) with MS were identified from the linked NHIS-NHIRD for 2013. The mean age was 59.79 (SD: ±12.85) years, with 56.47 (± 12.79) years for men and 63.67 (± 11.79) years for women (Table 1). Among the MS patients, 91.76% had hypertension, 69.18% had hyperlipidemia, 65.23% had BMI ≥ 27 kg/m^2^, and 61.41% had diabetes. 19.59% and 1.31% of patients were comorbid with osteoarthritis and depression, respectively, at the baseline. The comorbidities of interest occurred more frequently among women compared with men, except for diabetes and depression (Table 1).

**Table 1:** Baseline characteristics of patients with metabolic syndrome (overall and stratified by gender)

Baseline characteristics	Total population (*n* = 837)	Male (*n* = 451)	Female (*n* = 386)	*p*-value
**Demographics**				
Age at index date (years), mean (SD)	59.79 (12.85)	56.47 (12.79)	63.67 (11.79)	< 0.05
< 55	34.17	44.79	21.76	< 0.05
55 − 64	29.15	29.27	29.02	
65 − 74	23.42	16.63	31.35	
≥ 75	13.26	9.31	17.88	
**Socioeconomic and healthy behaviors, %**				
Marital status				< 0.05
Single	7.41	10.64	3.63	
Married	71.33	78.94	62.44	
Divorced or widowed	21.27	10.42	33.94	
Education level				< 0.05
Primary or below	49.46	44.57	55.18	
High school	20.67	26.16	14.25	
College/university or above	29.63	29.27	30.05	
Unknown	0.24	0.00	0.52	
Monthly household income (NTD^a^)				< 0.05
< 30,000	26.40	21.51	32.12	
30,000–69,000	33.09	33.92	32.12	
≥ 70,000	23.78	29.27	17.36	
Unknown	16.73	15.30	18.39	
Smoking history				< 0.05
Ever	19.59	33.70	3.11	
Never	17.20	30.60	1.55	
Unknown	63.20	35.70	95.34	
Betel nut chewing history				< 0.05
Ever	24.49	40.80	5.44	
Never	75.51	59.20	94.56	
**Comorbidity (yes), %**				
Hypertension	91.76	88.47	95.60	< 0.05
Diabetes	61.41	59.42	63.73	0.20
Hyperlipidemia	69.18	65.63	73.32	< 0.05
Osteoarthritis	19.59	12.20	28.24	< 0.05
Depression	1.31	0.89	1.81	0.36
**BMI (kg/m** ^**2**^ **), %**			< 0.05	
< 27	34.77	30.38	39.90	
27 − 30	38.83	41.02	36.27	
≥ 30	26.40	28.60	23.83	
**All-cause death, %**	12.54	10.64	14.77	0.07

The index date was defined as the date of a study subject being confirmed as having metabolic syndrome [i.e., had any three of the following criteria: (1) BMI ≥ 27 kg/m^2^, (2) hypertension, (3) diabetes, and (4) hyperlipidemia]

BMI: body mass index; SD: standard deviation; NTD: New Taiwan dollar

^a^The exchange rate between USD and NTD was 1:30.98 in 2023

During a mean follow-up period of 8.28 (± 1.35) years, the most frequent complications were nephropathy (i.e., 6.49 events per 100 person-years), followed by retinopathy (2.64), CVDs (2.47), neuropathy (2.08), and peripheral vascular diseases (2.01) (Table 2). In the 9 years of follow-up, the cumulative incidence of microvascular complications ranged from 16.7% (neuropathy) to 44.3% (nephropathy) and that of macrovascular conditions ranged from 9.7% (stroke) to 20.1% (CVDs) (Supplementary Fig. 1). The incidence rates of CVDs, stroke, peripheral vascular diseases, nephropathy, and cancers were significantly higher among elderly patients (aged ≥ 65 years) compared with their counterparties (all *p*-values < 0.05) (Table 2).

**Table 2:** Incidence rates of complications of interest among patients with metabolic syndrome (overall and stratified by gender and age)

Incidence rate (per 100 person-years)	Total population	Sex			Age (years)		
		Female	Male	*p*-value	< 65	≥ 65	*p*-value
Cardiovascular diseases	2.47	2.27	2.65	0.32	1.79	3.77	< 0.05
Cerebrovascular diseases	1.13	1.50	0.82	< 0.05	0.58	2.14	< 0.05
Peripheral vascular diseases	2.01	2.36	1.72	0.07	1.61	2.74	< 0.05
Nephropathy	6.49	6.59	6.40	0.78	5.29	8.87	< 0.05
Retinopathy	2.64	3.25	2.14	< 0.05	2.46	2.95	0.24
Neuropathy	2.08	2.42	1.80	0.08	2.14	1.97	0.66
Acute metabolic complications^a^	0.17	0.15	0.18	0.77	0.13	0.23	0.36
Other obesity-related medical conditions^b^	0.72	1.03	0.47	< 0.05	0.65	0.86	0.33
Cancers	1.53	1.46	1.59	0.67	1.22	2.11	< 0.05
MS-related cancers^c^	0.64	0.87	0.44	< 0.05	0.44	0.98	< 0.05
Other cancers^d^	0.84	0.55	1.10	< 0.05	0.74	1.02	0.23
All-cause death	1.51	1.79	1.28	0.09	0.49	3.45	< 0.05

MS: metabolic syndrome

^a^Acute metabolic complications included diabetic ketoacidosis and hyperosmolar hyperglycemic syndrome

^b^Other obesity-related medical conditions included sleep apnea, bariatric surgery, and knee replacement therapy

^c^MS-related cancers included liver, colorectal, bladder, pancreatic, endometrial, and breast postmenopausal cancers

^d^Other cancers included solid tumors (excluding MS-related cancers mentioned above), leukemia, and lymphoma

**Fig. 1:**
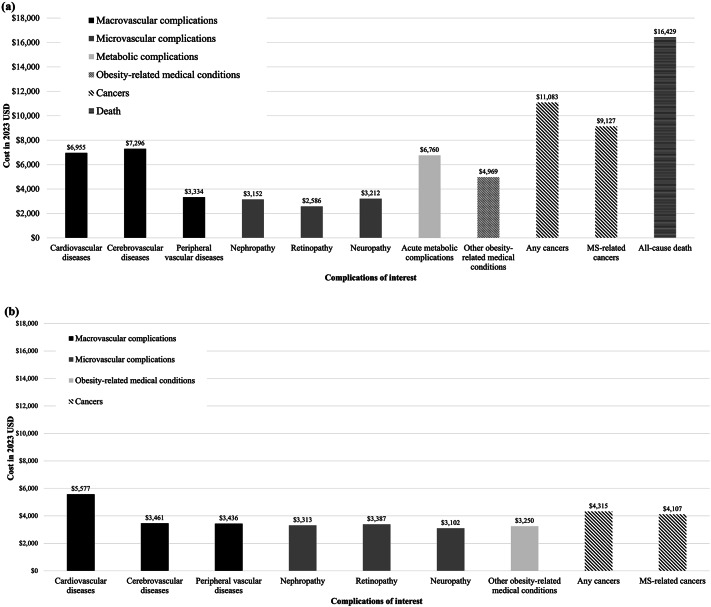
**a** Event-year and **b** annual state-year costs (per person) for metabolic syndrome-related complications of interest. MS, metabolic syndrome. (1) Acute metabolic complications included diabetic ketoacidosis and hyperosmolar hyperglycemic syndrome. (2) Other obesity-related medical conditions included sleep apnea, bariatric surgery, and knee replacement therapy. (3) MS-related cancers included liver, colorectal, bladder, pancreatic, endometrial, and breast postmenopausal cancers. Any cancers included MS-related and other cancers (e.g., solid tumors, lymphoma). (4) Event-year costs were measured as the healthcare costs in the year when the complication occurred. Annual state-year costs were the average annual costs in subsequent years following the event occurrence

Deaths resulted in enormous expenditure, with an average annual cost of USD 16,429 per person [Fig. 1(a)]. The event year costs of MS-related and all cancers (USD 9,127−11,083 per person), cerebrovascular diseases (USD 7,296 per person), CVDs (USD 6,955 per person), acute metabolic complications (USD 6,760 per person), other obesity-related medical conditions (USD 4,969 per person), and microvascular complications (USD 2,586−3,152 per person; these complications include nephropathy, retinopathy, and neuropathy, which are frequently seen in MS patients, as shown in Table 2) are presented in Fig. 1a. Figure 1b shows the annual state-year costs following the occurrence of complication events. One year after event occurrence, the cost of CVDs was USD 5,577 per person and the costs of the other complications were USD 3,102−4,315 per person. Compared with the healthcare costs of patients without any complications, those of patients who developed MS-related complications were higher during the study follow-up (Supplementary Fig. 2). Annual costs increased when CVDs or cerebrovascular diseases occurred [i.e., the cost in “Year 1”, Supplementary Fig. 2(a)], whereas the event and subsequent costs remained relatively stable over 6 years of follow-up when microvascular complications occurred [Supplementary Fig. 2(b)].

The cost multipliers obtained from the GEE model are presented in Table 3 (primary analysis) and Supplementary Tables 2−6 (sensitivity analyses). The baseline annual healthcare cost was USD 287.64 (USD 201.10−411.46) for a 55-year-old female with BMI < 27 kg/m^2^ and no comorbidities or established complications. The mean annual cost of an MS patient was significantly increased by the presence of clinical conditions other than those for the base case, namely being older than 55 years old (i.e., baseline costs multiplied by 1.20−1.39 for patients aged ≥ 55 years), having any comorbidities (costs multiplied by 1.20 [osteoarthritis]−2.08 [depression]), and developing an MS-related complication (costs multiplied by 1.23 [peripheral vascular diseases]−3.73 [other cancers]). The cost of MS patients who died increased in the year of death by a factor of 1.92 (1.50−2.46). In the sensitivity analysis where only the cause of death was specified, deaths attributable to other causes, CVDs, and MS-related cancers increased annual costs by a factor of 1.68 (1.42−2.00), 1.57 (1.19−2.08), and 1.28 (0.84−1.94), respectively (Supplementary Table 2). Moreover, having BMI ≥ 35 kg/m^2^ (morbid obesity) significantly increased the annual cost by a factor of 1.58 (1.30−1.91) (Supplementary Table 4). The results of sensitivity analyses that eliminated variables with unknown data (Supplementary Tables 5 and 6) are consistent with the primary findings (Table 3), supporting the robustness of this study.

**Table 3:** Cost multipliers for clinical characteristics of patients with metabolic syndrome (*n* = 837)

Characteristics	Multipliers	95% CIs	
Annual baseline healthcare cost (2023 USD), mean (95% CI)	287.64	201.10	411.46
**Demographics**			
Age at the index date (years) (ref.: < 55)			
55 − 64	1.20	1.08	1.35
65 − 74	1.35	1.18	1.55
≥ 75	1.39	1.18	1.63
Sex (ref.: female)	1.02	0.89	1.16
**Socioeconomic status and healthy behaviors**			
Education (ref.: primary or below)			
High school	0.99	0.87	1.12
College/university or above	0.92	0.84	1.02
Unknown	1.28	0.73	2.23
Marital status (ref.: single)			
Married	1.04	0.85	1.28
Divorced/widowed	1.13	0.90	1.41
Monthly household income (ref.: < NTD30,000^a^)			
NTD30,000 − 69,999	0.97	0.86	1.08
≥ NTD70,000	0.93	0.82	1.06
Unknown	0.93	0.81	1.07
Smoking status (ref.: never)			
Ever	0.89	0.77	1.03
Unknown	0.98	0.83	1.15
Betel nut chewing status (ref.: never)			
Ever	1.06	0.93	1.22
Body mass index (kg/m^2^) (ref.: < 27)^b^			
27 − 29.9 (mild obesity)	1.03	0.93	1.15
≥ 30 (moderate or morbid obesity)	1.10	0.98	1.25
**Comorbidity (ref.: none)**			
Hypertension^b^	1.62	1.34	1.95
Diabetes^b^	1.61	1.47	1.77
Hyperlipidemia^b^	1.33	1.20	1.46
Osteoarthritis	1.20	1.07	1.34
Depression	2.08	1.42	3.05
**Complications (event-year) (ref.: none)**			
Cardiovascular diseases	2.59	2.18	3.07
Cerebrovascular diseases	2.10	1.63	2.70
Peripheral vascular diseases	1.23	1.05	1.44
Nephropathy	1.27	1.17	1.39
Retinopathy	1.24	1.11	1.37
Neuropathy	1.27	1.11	1.46
Acute metabolic complications^c^	1.93	1.18	3.14
Other obesity-related medical conditions^d^	2.40	1.84	3.14
Cancers (ref.: none)			
MS-related cancers^e^	2.75	2.05	3.69
Other cancers^f^	3.73	2.81	4.97
**Complications (state-year) (ref.: none)**			
Cardiovascular diseases	1.63	1.39	1.92
Cerebrovascular diseases	1.10	0.89	1.37
Peripheral vascular diseases	1.11	0.96	1.28
Nephropathy	1.18	1.08	1.29
Retinopathy	1.22	1.09	1.36
Neuropathy	1.12	0.98	1.29
Other obesity-related medical conditions^d^	1.28	1.01	1.63
Cancers (ref.: none)			
MS-related cancers^e^	1.67	1.28	2.17
Other cancers^f^	2.26	1.63	3.13
**All-cause death (ref.: none)**	1.92	1.50	2.46

*Implications and illustrative example*: (1) Given the insignificant effects of sex, educational level, marital status, monthly household income, smoking status, betel nut chewing status, BMI, cerebrovascular diseases, peripheral vascular diseases, and neuropathy in state years, the cost multipliers of these characteristics in the future application of cost estimation can be regarded as 1 (implying no cost impact). (2) Example: The annual cost for an MS patient who is male and aged 60 years and had hypertension, diabetes, and BMI ≥ 30 kg/m^2^ at baseline and developed a cardiovascular disease (event-year) is USD 2,282 (i.e., USD 287.64 [baseline cost] × 1 [cost multiplier for male] × 1.2 [cost multiplier for 55–64 years] × 1.62 [cost multiplier for the presence of hypertension at baseline] × 1.61 [cost multiplier for the presence of diabetes at baseline] × 1 [BMI] × 2.59 [cost multiplier for developing a cardiovascular disease])

BMI: body mass index; MS: metabolic syndrome

^a^The exchange rate between USD and NTD was 1:30.98 in 2023

^b^MS-related risk factors included BMI, hypertension, hyperlipidemia and diabetes

^c^Acute metabolic complications included diabetic ketoacidosis and hyperosmolar hyperglycemic syndrome

^d^Other obesity-related medical conditions included sleep apnea, bariatric surgery, and knee replacement therapy

^e^MS-related cancers included liver, colorectal, bladder, pancreatic, endometrial, and breast postmenopausal cancers

^f^Other cancers included any malignancy (e.g., lymphoma and leukemia) except for malignant neoplasm of skin and MS-related cancers mentioned above

## Discussion

To the best of our knowledge, this study is the first to investigate the health economic burden across a wide range of incident MS-related complications (i.e., macro-/microvascular complications, cancers, obesity-related medical conditions) with a comprehensive adjustment for baseline patient characteristics (i.e., demographics, socioeconomic status, and concurrent comorbidities) over 9 years of follow-up. We found that incident cancers, CVDs, and other obesity-related medical conditions were major cost drivers in populations with MS, increasing medical expenditures by 140–273% in the year when the event developed (Table 3). This economic burden remained substantial over the years after the occurrence. The overall cost burden caused by more frequent microvascular complications cannot be ignored even though the cost impact of these complications is lower than that of other MS-related complications (e.g., CVDs). In addition, comorbid depression in patients with MS can contribute to an excess cost burden.

### Cancers, CVDs, and obesity-related medical conditions as top cost drivers of MS complications

Cancers [21, 25], CVDs [17, 26], and obesity-related medical conditions [19, 20] have been recognized as important complications as MS evolves. However, compared with CVDs, there is a lack of evidence regarding the economic burden of cancers and obesity-related medical conditions in MS populations. The present study adds supporting evidence showing that MS patients who developed cancers had a nearly 2- to 3-fold increase in annual medical costs in the event year and in subsequent years, regardless of MS/non-MS-related cancers. This cost impact can be explained by expensive cancer therapies and the frequent outpatient visits or inpatient stays required in oncology care [27]. Moreover, our results revealed an excess economic burden attributable to obesity-related medical conditions (e.g., sleep apnea, knee replacement) in MS individuals (i.e., 2.4-fold increase in medical expenditures), which is consistent with previous findings for patients with sleep apnea [28] and obesity [19, 29]. These results were expected because the obesity-related medical conditions identified in this study require costly medical procedures and indicate a group of patients who typically have high levels of BMI or are at risk of obesity, which are clinical features linked to excess healthcare utilization or consumption (e.g., long length of hospital stay [19, 29]). In addition, we found a larger cost burden caused by the development of CVDs and considerable maintenance costs in the following (state) years needed for continuous treatments to lower recurrence risks or rehabilitation to alleviate CVD-related disabilities. This economic burden is consistent with findings from previous studies, although the magnitude of the cost impact varies with the target population (e.g., MS populations [6, 8], non-diabetic MS adults [10], hypertensive patients with MS [30]), study follow-up period (e.g., 5 years [6, 10], 10 years [8]), and methodology (e.g., multivariable model [6, 10], simulation [8], prevalence-based model [30]). Nevertheless, the considerable economic burden attributed to the development of costly cancers and CVDs or obesity-related medical conditions in MS populations suggests the importance of early prevention to avert the occurrence of MS and its costly complications and thus reduce the substantial cost of follow-up care.

### Potentially considerable overall cost burden of microvascular complications

 Although MS is known to increase the risk of microvascular complications [17, 31], the economic burden of microvascular complications in MS populations has not been studied. We thus analyzed several clinically meaningful microvascular events (i.e., retinopathy, neuropathy, and nephropathy) and found a nearly 1.2-fold increase in annual medical costs when these complications occurred. The increased costs caused by microvascular complications in the following years (1.12−1.22-fold increase) can be explained by the additional treatments or medical interventions and procedures required during the chronic progression of these complications (e.g., from monitoring without treatments to receiving treatments, or from non-dialysis to dialysis-dependent [32]). Of note, the cost impact of microvascular complications in MS individuals and patients with type 2 diabetes is generally comparable (i.e., cost multipliers in event year [state years]: 1.24−1.27 [1.12−1.22] in MS populations versus 1.37−1.49 [1.13−1.18] in diabetic patients [12]). This is supported by the similarity in disease etiology between MS and diabetes populations [33, 34]. Nevertheless, it is worth noting that total healthcare expenditures attributable to microvascular complications could be considerable given a larger number of MS individuals developing microvascular complications (supported by high incidence rates in this study; Table 2), even though the cost impact of microvascular complications was lower than that of macrovascular complications. Hence, these results indicate the need for the early prevention of microvascular complications in MS populations to minimize the overall financial burden caused by these complications, and highlight the importance of including these complications in economic simulation analyses when identifying cost-effective intervention or treatment strategies in these populations.

### Depression, BMI, and other significant risk factors with certain cost impacts

This study also analyzed the cost impacts of a series of well-known comorbidities and risk factors associated with MS. The identified cost drivers deserve clinical attention. Specifically, we are the first to determine the cost burden of comorbid depression in MS populations. Similar to previous studies of other patient populations (i.e., type 2 diabetes [12], obesity [35], and CVDs [36]), a considerable cost impact of depression in MS individuals (i.e., cost multiplier: 2.08) was found in this study. Moreover, our results suggest the potential excess cost burden related to increased BMI levels in MS populations, as supported by larger cost multipliers associated with higher BMI cut-off points (Table 3 and Supplementary Table 3). In particular, MS patients with morbid obesity (BMI ≥ 35 kg/m^2^), who are qualified to receive bariatric surgery reimbursed by Taiwan’s NHI program, had a significant increase in annual healthcare expenditure (58% increase [95% CI: 30%, 91%], Supplementary Table 4) compared with that of patients with BMI levels < 35 kg/m^2^. A study from Finland showed that severely obese patients (BMI ≥ 35 kg/m^2^) had 40% (95% CI: 13%, 75%) higher age- and sex-adjusted direct medical costs compared with those of patients with normal weight (BMI 18.4–24.9 kg/m^2^) [37]. Finally, other MS-related risk factors (i.e., age) and comorbidities (e.g., hypertension, diabetes, hyperlipidemia, osteoarthritis) with significant cost impacts (i.e., cost multipliers > 1) were also identified in this study, which corroborate previously reported findings that showed higher healthcare costs for MS patients with a higher number of these risk factors/comorbidities [6, 7]. Hence, in addition to the complications caused by MS, the economic burden attributable to MS-related risk factors and comorbidities cannot be overlooked, highlighting the need for clinical attention to concurrent risk factors (e.g., morbid obesity) and comorbidities (e.g., depression) in MS populations.

### Study limitations

First, due to the unavailability of laboratory (e.g., lipid profiles) and anthropometric (e.g., waist circumference) data, we applied the presence of disease diagnosis (e.g., hyperlipidemia), the use of associated treatments (e.g., lipid-lowering agents), and/or BMI level [16] as criteria to identify MS individuals. In this regard, our study population may represent more severe MS cases (minor MS is generally determined based on only abnormal laboratory values or waist circumference measures) and thus might have larger healthcare expenditures attributable to the treatments required for established clinical conditions and diseases (e.g., glucose-lowering agents for type 2 diabetes). Second, the number of patients with medical procedures of interest (e.g., bariatric surgery and knee replacement) was limited, which might have affected the statistical power to differentiate the significant impact of individual clinical conditions. Third, indirect costs were not considered in this economic analysis due to data unavailability. Lastly, this study targeted an adult population (aged 18 years and above). However, given the rapidly increasing obesity in children and adolescent populations, future research on MS patients from younger populations is warranted.

In conclusion, this longitudinal economic analysis corroborated the substantial cost impacts associated with incident complications, MS-related risk factors, and comorbidities in individuals with MS, which not only highlights the urgent need for the prevention of MS and associated complications and risk factors during its progression, but also suggests the need for comprehensive and continuous management for this population. This study provides cost estimates of a wide range of incident complications, MS-related risk factors, and comorbidities in individuals with MS, which are essential for the parameterization of economic simulation models to determine long-term health and economic outcomes of MS populations, identify cost-effective interventions strategies, and ultimately inform clinical and policy efforts for improving healthcare in this population.

### Electronic supplementary material

Below is the link to the electronic supplementary material.

Supplementary Material 1

## Data Availability

The datasets generated and analyzed in the current study are not publicly available due to data privacy issues but are available from the corresponding author on reasonable request.

## Abbreviations

BMI: Body mass index
CIs: Confidence interval
CVDs: Cardiovascular diseases
GEE: Generalized estimating equation
MS: Metabolic syndrome
NHIRD: National Health Insurance Research Database
NHIS: National Health Interview Survey
SDs: Standard deviations
STROBE: Strengthening the Reporting of Observational Studies in Epidemiology
USD: United States dollars
