# Supplementary material for: Longitudinal economic burden of incident complications among metabolic syndrome populations
Source: Cardiovasc Diabetol. 2024 Jul 10;23:246. doi: 10.1186/s12933-024-02335-7 (PMC11238381; doi:10.1186/s12933-024-02335-7)
Supplement: Supplementary file 1 — Supplementary Material 1 [file 12933_2024_2335_MOESM1_ESM.docx]

**Supplementary results**

- Supplementary Method 1: Detailed information of data sources
- Supplementary Method 2: Detailed description of event-year and state-year cost
- Supplementary Method 3: Detailed description of GEE model and the implication of study results
- Supplementary Table 1: Operational definitions of MS-related risk factors, comorbidities and complications of interest
- Supplementary Table 2: Cost multipliers for clinical characteristics of patients with metabolic syndrome (sensitivity analysis specifying cause of death)
- Supplementary Table 3: Cost multipliers for clinical characteristics of patients with metabolic syndrome (sensitivity analysis using BMI ≥ 30 kg/m^2^ as cut-off point for obesity)
- Supplementary Table 4: Cost multipliers for clinical characteristics of patients with metabolic syndrome (sensitivity analysis using BMI ≥ 35 kg/m^2^ as cut-off point for obesity)
- Supplementary Table 5. Cost multipliers for clinical characteristics of patients with metabolic syndrome (sensitivity analysis without smoking status, which accounted for more than 50% of unknown data)
- Supplementary Table 6. Cost multipliers for clinical characteristics of patients with metabolic syndrome (sensitivity analysis without clinical characteristics [i.e., smoking, education level, and monthly income] that had unknown data)
- Supplementary Table 7. The Strengthening the Reporting of Observational Studies in Epidemiology (STROBE) checklist
- Supplementary Figure1: Cumulative incidence rates of metabolic syndrome-related complications of interest
- Supplementary Figure 2: Crude annual healthcare costs (per person) of (a) macrovascular complications, (b) microvascular complications, and (c) other complications, compared with those of non-complications (“NC”) from baseline year (before complication occurred; Year 0) up to 6 years of follow-up (Years 1−6)

**Supplementary Method 1: Detailed information of data sources**

The National Health Interview Survey is a cross-sectional survey of a representative sample from each county or municipality in Taiwan implemented using a stratified multi-stage systematic sampling procedure (1). The survey includes self-reported personal characteristics, health behaviors, social economic status, and health measurements (e.g., height, weight, health utility). The National Health Insurance Research Database is a Taiwanese population-based longitudinal claims database of medical records of outpatient, inpatient, and emergency department visits, prescription drugs, and medical supplies. It covers more than 99% of Taiwan’s population given the mandatory and universal enrollment in Taiwan’s National Health Insurance (2).

References:

1. Health Promotion Administration, Ministry of Health and Welfare, Taiwan. National Health Interview Survey (NHIS). Accessed 3 October 2022. Available from <https://www.hpa.gov.tw/Pages/Detail.aspx?nodeid=1077&pid=6198>.
2. Hsieh CY, Su CC, Shao SC, et al. Taiwan's National Health Insurance Research Database: past and future. Clin Epidemiol. 2019; 11: 349-58.

**Supplementary Method 2: Detailed description of event-year and state-year cost**

Event-year costs reflect the healthcare resources allocated to an acute episode when it occurred and state-year costs reflect the subsequent care and management during the rest of patient life following the event occurrence. In this study, the event-year costs were the healthcare expenditures in the year when a metabolic syndrome-related complication occurred and the state-year costs were the average annual costs from the years after the event year.

**Supplementary Method 3. Detailed description of GEE model and the implication of study results**

The generalized estimating equation (GEE) analysis was chosen because of potential within-person correlations (non-dependence) in repeated (or autocorrelated) data (i.e., annual healthcare costs, occurrence of complications) over time. Also, log transformation was applied to deal with the skewed healthcare expenditure data for normality. In the GEE model, baseline patient characteristics and comorbidities were treated as time-invariant exploratory variables and the complications of interest were treated as time-varying variables. The collinearity between exploratory variables in the GEE was examined using the variance inflation factor (VIF), where a VIF value of < 10 indicates non-collinearity.

The base case of the GEE model was a single female aged less than 55 years old, no smoking or chewing betel nut history, with body mass index< 27 kg/m^2^, receiving primary school education or below, having the average of monthly household income < $30,000 (New Taiwan Dollars), and no comorbidities or established complications. If a patient had any characteristics other than those for the base case, the mean annual healthcare cost was estimated as the baseline cost multiplied by the product of the cost multipliers for each characteristic of that patient.

**Supplementary Table 1. Operational definitions of MS-related risk factors, comorbidities and complications of interest**

|  | ICD-9-CM (for comorbidities)/PCS (for procedures) | ICD-10-CM (for comorbidities)/PCS (for procedures) | File(s) used in NHIRD |
| --- | --- | --- | --- |
| **MS-related risk factors** |  |  |  |
| Hypertension^1^ | 401-405 | I10, I11, I12, I13, I15, I16 | OPD, IPD |
| Diabetes^2^ | 250 | E10, E11 | OPD, IPD |
| Hyperlipidemia^1^ | 272 | E78 | OPD, IPD |
| **Comorbidities** |  |  |  |
| Osteoarthritis^3,4^ | 715 | M15-M19 | OPD, IPD |
| Depression^5^ | 296.2, 296.3 | F32, F33 | OPD, IPD |
| **Complications** |  |  |  |
| Macrovascular and microvascular diseases | | | |
| Cardiovascular diseases^6^ |  |  | IPD, ER |
| Acute myocardial infarction | 410 | I21-I23 |  |
| Ischemic heart disease | 411-414 | I20, I24, I25 |  |
| Heart failure | 428 | I50 |  |
| Cerebrovascular diseases^6^ | 431-435 | I60-I63, I65, I66, G45, G46, I67.84 | IPD, ER |
| Peripheral vascular diseases^6-8^ | 040.0, 250.7, 440, 442.3, 443.81, 443.9, 892.1, 444.22, 785.4, 707.1, V49.7 (with procedure code 84.1) | A48.0, Exx.51, Exx.52, Exx.59 (xx=08-11, 13), I70.2-I70.7, I72.4, I73.9, I74.3, L97, S91.3, Z89 (with procedure code 0Y6) | OPD, IPD |
| Nephropathy^6^ | 250.4, 580-583, 585, 586, 593.9, 791, V42.0 | Exx.2 (xx=08-11, 13), N00.0-N00.9, N01.0-N01.9, N03-N05, N08, N17-N19, R80.9, Z94.0 | OPD, IPD |
| Retinopathy^6^ | 250.5, 361.0, 362.02, 362.04-362.07, 362.1, 362.53, 362.81, 362.82, 362.83, 369, 379.23 | Exx.3 (xx=08-11, 13), H33.0, H35.0, H35.35, H35.6, H35.8, H35.9, H43.1, H54.0, H54.4, | OPD, IPD |
| Neuropathy^6^ | 250.6, 354, 355, 356.9, 357.2, 358.1, 951.0, 951.1, 951.3 | Exx.4 (xx=08-11, 13), G56, G57, G60.9, G73.3, S04 | OPD, IPD |
| Metabolic complications |  |  |  |
| Hyperglycemia (including DKA and HHS)^6^ | 250.1, 250.2 | Exx.0 (xx=08-11, 13), Exx.1 (xx=08-11, 13), Exx.64 (xx=08-11, 13) | IPD, ER |
| Other obesity-related medical conditions | | | |
| Sleep apnea^9-11^ | 780.51, 780.53, 780.57 | G47.3 | OPD, IPD |
| Bariatric surgery^11,12^ | 43.82, 43.89, 44.31, 44.38, 44.39, 44.68, 44.69, 44.95, 44.99 | 0DB60ZZ, 0DB63ZZ, 0DB64Z3, 0DB67ZZ, 0D1607A, 0D160JA, 0D160KA, 0D160ZA, 0D1687A, 0D168JA, 0D168KA, 0D168ZA, 0D16079, 0D1607A, 0D1607B, 0D1607L, 0D160J9, 0D160JA, 0D160JB, 0D160JL, 0D160K9, 0D160KA, 0D160KB, 0D160KL, 0D160Z9, 0D160ZA, 0D160ZB, 0D160ZL, 0D16479, 0D1647A, 0D1647B, 0D1647L, 0D164J9, 0D164JA, 0D164JB, 0D164JL, 0D164K9, 0D164KA, 0D164KB, 0D164KL, 0D164Z9, 0D164ZA, 0D164ZB, 0D164ZL, 0D16879, 0D1687A, 0D1687B, 0D1687L, 0D168J9, 0D168JA, 0D168JB, 0D168JL, 0D168K9,0D168KA, 0D168KB, 0D168KL, 0D168Z9, 0D168ZA, 0D168ZB, 0D168ZL, 0D760DZ, 0D760ZZ, 0D763DZ, 0D763ZZ, 0D764DZ, 0D764ZZ, 0D767DZ, 0D767ZZ, 0D768DZ, 0D768ZZ, 0DF60ZZ, 0DF63ZZ, 0DF64ZZ, 0DF67ZZ, 0DF68ZZ, 0DM60ZZ, 0DM64ZZ, 0DQ60ZZ, 0DQ63ZZ, 0DQ64ZZ, 0DQ67ZZ, 0DQ68ZZ, 0DU607Z, 0DU60JZ, 0DU60KZ, 0DU647Z, 0DU64JZ, 0DU64KZ, 0DU677Z, 0DU67JZ, 0DU67KZ, 0DU687Z, 0DU68JZ, 0DU68KZ, 0DV60CZ, 0DV60DZ, 0DV60ZZ, 0DV63CZ, 0DV63DZ, 0DV63ZZ, 0DV64CZ, 0DV64DZ, 0DV64ZZ, 0DV67ZZ, 0DV68ZZ, 0DW64CZ, 0DL60CZ, 0DL60DZ, 0DL60ZZ, 0DL63CZ, 0DL63DZ, 0DL63ZZ, 0DL64CZ, 0DL64DZ, 0DL64ZZ, 0DL67DZ, 0DL67ZZ, 0DL68DZ, 0DL68ZZ, 0DL70CZ, 0DL70DZ, 0DL70ZZ, 0DL73CZ, 0DL73DZ, 0DL73ZZ, 0DL74CZ, 0DL74DZ, 0DL74ZZ, 0DL77DZ, 0DL77ZZ, 0DL78DZ, 0DL78ZZ, 0DP643Z, 0DP64CZ, 0DQ60ZZ, 0DQ63ZZ, 0DQ64ZZ, 0DQ67ZZ, 0DQ68ZZ, 0DQ70ZZ, 0DQ73ZZ, 0DQ74ZZ, 0DQ77ZZ, 0DQ78ZZ, 0DW64CZ, 0DW68CZ, 0DY60Z0, 0DY60Z1, 0DY60Z2, 3E0G3GC | IPD |
| Knee replacement^13^ | 64164B, 64202B, 97805K, 97806A, 97807B | | IPD |
| Any cancers |  |  |  |
| MS-related cancers^14-16^ |  |  | OPD, IPD |
| Liver cancer | 155 | C22 |  |
| Colorectal cancer | 153, 154 | C18-C21 |  |
| Bladder cancer | 188 | C67 |  |
| Pancreatic cancer | 157 | C25 |  |
| Endometrial cancer | 182 | C54 |  |
| Breast cancer | 174 | C50 |  |
| Other cancers^16^ | 140-152, 156, 158-172, 175-181, 183-187, 189-195.8, 200-208, 238.6 | C00-C17, C23, C24, C26, C30-C34, C37-C41, C43, C45-C49, C51-C52, C55-C58, C60-C66, C68-C76, C81-C85, C88, C90-C97 | OPD, IPD |

Abbreviations: ATC, Anatomical Therapeutic Chemical; DKA, diabetic ketoacidosis; ER, emergency department; HHS, hyperosmolar hyperglycemic state; ICD-9-CM, International Classification of Diseases, Ninth Revision, Clinical Modification; ICD-10-CM, International Classification of Diseases, Tenth Revision, Clinical Modification; ICD-9-PCS, ICD-9 Procedure Coding System; ICD-10-PCS, ICD-10 Procedure Coding System; IPD, inpatient department; MS, metabolic syndrome; NHIRD, National Health Insurance Research Database; OPD, outpatient department.

Notes:

1. In additional to disease diagnosis codes, hypertension, diabetes, and hyperlipidemia were also ascertained according to the use of relevant medications defined using the Anatomical Therapeutic Chemical (ATC) system (i.e., C02, C03, C07A, C07BB, C07CB, C08CA, C08D, and C09 for hypertension, A10 for diabetes, and C10 for hyperlipidemia).
2. Instead of ICD-9-PCS and ICD-10-PCS, the reimbursement codes of Taiwan’s National Health Insurance were applied to measure the receipt of knee replacement.

**References:**

1. *Cardiovasc Diabetol.* 2019;18(1):120.
2. *JAMA Netw Open*. 2021;4(12):e2138775.
3. *Front Med (Lausanne).* 2020;7:392.
4. "National Arthritis Data Workgroup ICD-9-CM diagnostic codes for arthritis and other rheumatic conditions." Centers for Disease Control and Prevention, Arthritis Program, National Arthritis Data Workgroup.
5. *J Formos Med Assoc*. 2022;121(10):2001-2011.
6. *J Diabetes Complications*. 2017;31(6):1007-1013.
7. *Diabetes Care*. 2018;41(5):917-928.
8. *Diabetes Care*. 2023;46(1):149–155
9. *Sci Rep.* 2021;11(1):765*.*
10. *Family Medicine & Primary Medical Care*. 2019;34(1):2-8.
11. Conversion of International Classification of Diseases, Ninth Revision, Clinical Modification (ICD-9-CM) to International Classification to Diseases, Tenth Revision, Clinical Modification (ICD-10-CM). National Health Insurance Administration, Ministry of Health and Welfare. <https://www.nhi.gov.tw/Content_List.aspx?n=C1C92AB9ED30A9FD&topn=23C660CAACAA159D>. Accessed on 30 Nov 2022.
12. *Obes Surg.* 2021;(9):4015-4023.
13. Reimbursement code. National Health Insurance Administration, Ministry of Health and Welfare. <https://info.nhi.gov.tw/INAE5000/INAE5001S01>. Accessed on 30 Nov 2022.
14. *Diabetes Care*. 2012;35(11):2402–2411
15. *European Journal of Cancer*. 2008;44(2):293–297.
16. *Med Care.* 2005;43(11):1130–1139.

**Supplementary Table 2. Cost multipliers for clinical characteristics of patients with metabolic syndrome (sensitivity analysis specifying cause of death)**

| **Study variables** | | **Multiplier** | **95% CI** | |
| --- | --- | --- | --- | --- |
| Baseline annual healthcare cost (2023 USD), mean (95% CI) | | 295.78 | 206.87 | 422.88 |
| Demographics | |  |  |  |
| Age at the index date (years) (ref.: < 55) | |  |  |  |
|  | 55-64 | 1.18 | 1.05 | 1.31 |
|  | 65-74 | 1.29 | 1.12 | 1.47 |
|  | ≥ 75 | 1.22 | 1.03 | 1.44 |
| Sex (ref.: female) | | 1.02 | 0.89 | 1.16 |
| Socioeconomic status and healthy behaviors | |  |  |  |
| Education (ref.: Primary or below) | |  |  |  |
|  | High school | 0.99 | 0.88 | 1.12 |
|  | College/university or above | 0.92 | 0.84 | 1.02 |
|  | Unknown | 1.45 | 0.84 | 2.52 |
| Marital status (ref.: single) | |  |  |  |
|  | Married | 1.05 | 0.87 | 1.28 |
|  | Divorced/widowed | 1.14 | 0.91 | 1.41 |
| Monthly household income (ref.: < NTD30,000^§^) | |  |  |  |
|  | NTD30,000−69,999 | 0.96 | 0.86 | 1.08 |
|  | ≥ NTD70,000 | 0.93 | 0.82 | 1.06 |
|  | Unknown | 0.93 | 0.81 | 1.07 |
| Smoking status (ref.: never) | |  |  |  |
| Ever | | 0.89 | 0.76 | 1.03 |
| Unknown | | 0.98 | 0.84 | 1.16 |
| Betel nut chewing status (ref.: never) | |  |  |  |
|  | Ever | 1.05 | 0.92 | 1.20 |
| Body mass index (kg/m^2^) (ref.: < 27) ^\|\|^ | |  |  |  |
| 27−< 29.9 (mild obesity) | | 1.02 | 0.92 | 1.14 |
| ≥ 30 (moderate or morbid obesity) | | 1.10 | 0.97 | 1.24 |
| Comorbidity (ref.: none) | |  |  |  |
| Hypertension^\|\|^ | | 1.58 | 1.31 | 1.90 |
| Diabetes^\|\|^ | | 1.57 | 1.43 | 1.72 |
| Hyperlipidemia^\|\|^ | | 1.32 | 1.20 | 1.45 |
| Osteoarthritis | | 1.22 | 1.09 | 1.37 |
| Depression | | 2.12 | 1.46 | 3.08 |
| Complications (event-year) (ref.: none) | |  |  |  |
| Cardiovascular diseases | | 2.55 | 2.15 | 3.03 |
| Cerebrovascular diseases | | 2.06 | 1.60 | 2.66 |
| Peripheral vascular diseases | | 1.22 | 1.05 | 1.43 |
| Nephropathy | | 1.26 | 1.16 | 1.37 |
| Retinopathy | | 1.24 | 1.12 | 1.38 |
| Neuropathy | | 1.28 | 1.12 | 1.46 |
| Acute metabolic complications^*^ | | 1.85 | 1.09 | 3.12 |
| Other obesity-related medical conditions^†^ | | 2.39 | 1.84 | 3.09 |
| Cancers (ref.: none) | |  |  |  |
|  | MS-related cancers ^††^ | 2.77 | 2.07 | 3.71 |
|  | Other cancers^¶^ | 3.87 | 2.90 | 5.15 |
| Complications (state-year) (ref.: none) | |  |  |  |
| Cardiovascular diseases | | 1.64 | 1.40 | 1.92 |
| Cerebrovascular diseases | | 1.12 | 0.90 | 1.39 |
| Peripheral vascular diseases | | 1.11 | 0.96 | 1.28 |
| Nephropathy | | 1.20 | 1.09 | 1.31 |
| Retinopathy | | 1.24 | 1.11 | 1.38 |
| Neuropathy | | 1.14 | 0.99 | 1.30 |
| Other obesity-related medical conditions^†^ | | 1.28 | 1.02 | 1.62 |
| Cancers (ref.: none) | |  |  |  |
|  | MS-related cancers^††^ | 1.75 | 1.34 | 2.29 |
|  | Other cancers^¶^ | 2.42 | 1.75 | 3.36 |
| All-cause death (ref.: none) | |  |  |  |
| Fatal cardiovascular diseases | | 1.57 | 1.19 | 2.08 |
| Fatal MS-related cancers | | 1.28 | 0.84 | 1.94 |
| Other cause deaths | | 1.68 | 1.42 | 2.00 |

Abbreviations: BMI, body mass index; MS, metabolic syndrome.

Notes:

^*^ Acute metabolic complications included diabetic ketoacidosis and hyperosmolar hyperglycemic syndrome.

^†^ Other obesity-related medical conditions included sleep apnea, bariatric surgery, and knee replacement therapy.

^††^ MS-related cancers included liver, colorectal, bladder, pancreatic, endometrial, and breast postmenopausal cancers.

^¶^ Other cancers included any malignancy such as lymphoma and leukemia, except malignant neoplasm of skin and MS-related cancers mentioned above.

^§^ The exchange rate between USD and NTD was 1:30.98 in 2023.

^||^MS-related risk factors included BMI ≥ 27 kg/m^2^, and the presence of hypertension, hyperlipidemia and diabetes.

**Supplementary Table 3. Cost multipliers for clinical characteristics of patients with metabolic syndrome (sensitivity analysis using BMI ≥ 30 kg/m^2^ as cut-off point for obesity)**

| **Study variables** | | **Multiplier** | **95% CI** | |
| --- | --- | --- | --- | --- |
| Baseline annual healthcare cost (2023 USD), mean (95% CI) | | 297.26 | 209.89 | 420.99 |
| Demographics | |  |  |  |
| Age at the index date (years) (ref.: < 55) | |  |  |  |
|  | 55−64 | 1.20 | 1.07 | 1.34 |
|  | 65−74 | 1.35 | 1.18 | 1.54 |
|  | ≥ 75 | 1.38 | 1.18 | 1.62 |
| Sex (ref.: female) | | 1.02 | 0.89 | 1.16 |
| Socioeconomic status and healthy behaviors | |  |  |  |
| Education (ref.: Primary or below) | |  |  |  |
|  | High school | 0.99 | 0.87 | 1.12 |
|  | College/university or above | 0.92 | 0.84 | 1.02 |
|  | Unknown | 1.27 | 0.73 | 2.21 |
| Marital status (ref.: single) | |  |  |  |
|  | Married | 1.04 | 0.85 | 1.28 |
|  | Divorced/widowed | 1.13 | 0.90 | 1.41 |
| Monthly household income (ref.: < NTD30,000^§^) | |  |  |  |
|  | NTD30,000-69,999 | 0.97 | 0.86 | 1.08 |
|  | ≥ NTD70,000 | 0.93 | 0.82 | 1.06 |
|  | Unknown | 0.93 | 0.81 | 1.07 |
| Smoking status (ref.: never) | |  |  |  |
| Ever | | 0.89 | 0.76 | 1.03 |
| Unknown | | 0.98 | 0.83 | 1.15 |
| Betel nut chewing status (ref.: never) | |  |  |  |
|  | Ever | 1.07 | 0.93 | 1.22 |
| Body mass index (kg/m^2^) (ref.: < 30)^\|\|^ | |  |  |  |
| ≥ 30 (moderate or morbid obesity) | | 1.08 | 0.98 | 1.20 |
| Comorbidity (ref.: none) | |  |  |  |
| Hypertension^\|\|^ | | 1.60 | 1.34 | 1.93 |
| Diabetes^\|\|^ | | 1.60 | 1.46 | 1.75 |
| Hyperlipidemia^\|\|^ | | 1.32 | 1.20 | 1.46 |
| Osteoarthritis | | 1.20 | 1.07 | 1.34 |
| Depression | | 2.07 | 1.42 | 3.02 |
| Complications (event-year) (ref.: none) | |  |  |  |
| Cardiovascular diseases | | 2.59 | 2.18 | 3.08 |
| Cerebrovascular diseases | | 2.10 | 1.63 | 2.70 |
| Peripheral vascular diseases | | 1.23 | 1.05 | 1.44 |
| Nephropathy | | 1.27 | 1.17 | 1.39 |
| Retinopathy | | 1.24 | 1.11 | 1.37 |
| Neuropathy | | 1.27 | 1.11 | 1.46 |
| Acute metabolic complications^*^ | | 1.92 | 1.18 | 3.14 |
| Other obesity-related medical conditions^†^ | | 2.41 | 1.84 | 3.14 |
| Cancers (ref.: none) | |  |  |  |
|  | MS-related cancers ^††^ | 2.75 | 2.05 | 3.69 |
|  | Other cancers^¶^ | 3.73 | 2.80 | 4.96 |
| Complications (state-year) (ref.: none) | |  |  |  |
| Cardiovascular diseases | | 1.64 | 1.39 | 1.92 |
| Cerebrovascular diseases | | 1.10 | 0.89 | 1.37 |
| Peripheral vascular diseases | | 1.11 | 0.96 | 1.28 |
| Nephropathy | | 1.18 | 1.08 | 1.29 |
| Retinopathy | | 1.21 | 1.09 | 1.35 |
| Neuropathy | | 1.13 | 0.98 | 1.29 |
| Other obesity-related medical conditions^†^ | | 1.29 | 1.02 | 1.63 |
| Cancers (ref.: none) | |  |  |  |
|  | MS-related cancers^††^ | 1.67 | 1.28 | 2.17 |
|  | Other cancers^¶^ | 2.25 | 1.62 | 3.12 |
| All cause death (ref.: none) | | 1.92 | 1.50 | 2.46 |

Abbreviations: BMI, body mass index; MS, metabolic syndrome.

Notes:

^*^ Acute metabolic complications included diabetic ketoacidosis and hyperosmolar hyperglycemic syndrome.

^†^ Other obesity-related medical conditions included sleep apnea, bariatric surgery, and knee replacement therapy.

^††^ MS-related cancers included liver, colorectal, bladder, pancreatic, endometrial, and breast postmenopausal cancers.

^¶^ Other cancers included any malignancy such as lymphoma and leukemia, except malignant neoplasm of skin and MS-related cancers mentioned above.

^§^ The exchange rate between USD and NTD was 1:30.98 in 2023.

^||^MS-related risk factors included BMI ≥ 27 kg/m^2^, and the presence of hypertension, hyperlipidemia and diabetes.

**Supplementary Table 4. Cost multipliers for clinical characteristics of patients with metabolic syndrome (sensitivity analysis using BMI ≥ 35 kg/m^2^ as cut-off point for obesity)**

| **Study variables** | | **Multiplier** | **95% CI** | |
| --- | --- | --- | --- | --- |
| Baseline annual healthcare cost (2023 USD), mean (95% CI) | | 299.26 | 212.34 | 421.79 |
| Demographics | |  |  |  |
| Age at the index date (years) (ref.: < 55) | |  |  |  |
|  | 55−64 | 1.21 | 1.08 | 1.35 |
|  | 65−74 | 1.36 | 1.19 | 1.55 |
|  | ≥ 75 | 1.39 | 1.19 | 1.63 |
| Sex (ref.: female) | | 1.02 | 0.90 | 1.16 |
| Socioeconomic status and healthy behaviors | |  |  |  |
| Education (ref.: Primary or below) | |  |  |  |
|  | High school | 0.98 | 0.87 | 1.11 |
|  | College/university or above | 0.93 | 0.84 | 1.02 |
|  | Unknown | 1.27 | 0.73 | 2.19 |
| Marital status (ref.: single) | |  |  |  |
|  | Married | 1.05 | 0.86 | 1.28 |
|  | Divorced/widowed | 1.12 | 0.90 | 1.39 |
| Monthly household income (ref.: < NTD30,000^§^) | |  |  |  |
|  | NTD30,000−69,999 | 0.97 | 0.86 | 1.08 |
|  | ≥ NTD70,000 | 0.93 | 0.82 | 1.06 |
|  | Unknown | 0.94 | 0.82 | 1.07 |
| Smoking status (ref.: never) | |  |  |  |
| Ever | | 0.88 | 0.75 | 1.02 |
| Unknown | | 0.98 | 0.83 | 1.15 |
| Betel nut chewing status (ref.: never) | |  |  |  |
|  | Ever | 1.06 | 0.93 | 1.21 |
| Body mass index (kg/m^2^) (ref.: < 35)^\|\|^ | |  |  |  |
| ≥ 35 (morbid obesity) | | 1.58 | 1.30 | 1.91 |
| Comorbidity (ref.: none) | |  |  |  |
| Hypertension^\|\|^ | | 1.60 | 1.33 | 1.92 |
| Diabetes^\|\|^ | | 1.58 | 1.44 | 1.72 |
| Hyperlipidemia^\|\|^ | | 1.33 | 1.21 | 1.46 |
| Osteoarthritis | | 1.20 | 1.07 | 1.34 |
| Depression | | 2.12 | 1.46 | 3.08 |
| Complications (event-year) (ref.: none) | |  |  |  |
| Cardiovascular diseases | | 2.58 | 2.17 | 3.07 |
| Cerebrovascular diseases | | 2.11 | 1.64 | 2.72 |
| Peripheral vascular diseases | | 1.22 | 1.05 | 1.43 |
| Nephropathy | | 1.27 | 1.17 | 1.39 |
| Retinopathy | | 1.24 | 1.12 | 1.38 |
| Neuropathy | | 1.27 | 1.11 | 1.45 |
| Acute metabolic complications^*^ | | 1.94 | 1.19 | 3.16 |
| Other obesity-related medical conditions^†^ | | 2.35 | 1.81 | 3.05 |
| Cancers (ref.: none) | |  |  |  |
|  | MS-related cancers ^††^ | 2.74 | 2.04 | 3.68 |
|  | Other cancers^¶^ | 3.75 | 2.82 | 4.98 |
| Complications (state-year) (ref.: none) | |  |  |  |
| Cardiovascular diseases | | 1.63 | 1.39 | 1.91 |
| Cerebrovascular diseases | | 1.11 | 0.90 | 1.39 |
| Peripheral vascular diseases | | 1.11 | 0.96 | 1.28 |
| Nephropathy | | 1.18 | 1.08 | 1.29 |
| Retinopathy | | 1.22 | 1.10 | 1.36 |
| Neuropathy | | 1.12 | 0.98 | 1.28 |
| Other obesity-related medical conditions^†^ | | 1.25 | 0.98 | 1.58 |
| Cancers (ref.: none) | |  |  |  |
|  | MS-related cancers^††^ | 1.66 | 1.27 | 2.16 |
|  | Other cancers^¶^ | 2.27 | 1.64 | 3.14 |
| All cause death (ref.: none) | | 1.92 | 1.50 | 2.46 |

Abbreviations: BMI, body mass index; MS, metabolic syndrome.

Notes:

^*^ Acute metabolic complications included diabetic ketoacidosis and hyperosmolar hyperglycemic syndrome.

^†^ Other obesity-related medical conditions included sleep apnea, bariatric surgery, and knee replacement therapy.

^††^ MS-related cancers included liver, colorectal, bladder, pancreatic, endometrial, and breast postmenopausal cancers.

^¶^ Other cancers included any malignancy such as lymphoma and leukemia, except malignant neoplasm of skin and MS-related cancers mentioned above.

^§^ The exchange rate between USD and NTD was 1:30.98 in 2023.

^||^MS-related risk factors included BMI ≥ 27 kg/m^2^, and the presence of hypertension, hyperlipidemia and diabetes.

**Supplementary Table 5. Cost multipliers for clinical characteristics of patients with metabolic syndrome (sensitivity analysis without smoking status, which accounted for more than 50% of unknown data)**

| **Study variables** | | **Multiplier** | **95% CI** | |
| --- | --- | --- | --- | --- |
| Baseline annual healthcare cost (2023 USD), mean (95% CI) | | 277.19 | 203.87 | 376.87 |
| Demographics | |  |  |  |
| Age at the index date (years) (ref.: < 55) | |  |  |  |
|  | 55−64 | 1.21 | 1.08 | 1.36 |
|  | 65−74 | 1.37 | 1.20 | 1.57 |
|  | ≥ 75 | 1.41 | 1.20 | 1.66 |
| Sex (ref.: female) | | 1.00 | 0.90 | 1.11 |
| Socioeconomic status and healthy behaviors | |  |  |  |
| Education (ref.: Primary or below) | |  |  |  |
|  | High school | 0.99 | 0.88 | 1.12 |
|  | College/university or above | 0.93 | 0.84 | 1.02 |
|  | Unknown | 1.28 | 0.74 | 2.24 |
| Marital status (ref.: single) | |  |  |  |
|  | Married | 1.04 | 0.85 | 1.27 |
|  | Divorced/widowed | 1.12 | 0.90 | 1.40 |
| Monthly household income (ref.: < NTD30,000^§^) | |  |  |  |
|  | NTD30,000−69,999 | 0.97 | 0.87 | 1.08 |
|  | ≥ NTD70,000 | 0.94 | 0.82 | 1.07 |
|  | Unknown | 0.93 | 0.82 | 1.07 |
| Betel nut chewing status (ref.: never) | |  |  |  |
|  | Ever | 1.04 | 0.93 | 1.17 |
| Body mass index (kg/m^2^) (ref.: < 27)^\|\|^ | |  |  |  |
| 27−< 29.9 (mild obesity) | | 1.04 | 0.93 | 1.16 |
| ≥ 30 (moderate or morbid obesity) | | 1.11 | 0.98 | 1.26 |
| Comorbidity (ref.: none) | |  |  |  |
| Hypertension^\|\|^ | | 1.62 | 1.34 | 1.96 |
| Diabetes^\|\|^ | | 1.60 | 1.46 | 1.76 |
| Hyperlipidemia^\|\|^ | | 1.33 | 1.20 | 1.46 |
| Osteoarthritis | | 1.20 | 1.07 | 1.34 |
| Depression | | 2.04 | 1.41 | 2.97 |
| Complications (event-year) (ref.: none) | |  |  |  |
| Cardiovascular diseases | | 2.60 | 2.19 | 3.09 |
| Cerebrovascular diseases | | 2.09 | 1.62 | 2.70 |
| Peripheral vascular diseases | | 1.23 | 1.05 | 1.44 |
| Nephropathy | | 1.27 | 1.17 | 1.38 |
| Retinopathy | | 1.24 | 1.11 | 1.37 |
| Neuropathy | | 1.27 | 1.11 | 1.45 |
| Acute metabolic complications^*^ | | 1.92 | 1.17 | 3.15 |
| Other obesity-related medical conditions^†^ | | 2.40 | 1.84 | 3.13 |
| Cancers (ref.: none) | |  |  |  |
|  | MS-related cancers ^††^ | 2.76 | 2.05 | 3.70 |
|  | Other cancers^¶^ | 3.73 | 2.81 | 4.96 |
| Complications (state-year) (ref.: none) | |  |  |  |
| Cardiovascular diseases | | 1.64 | 1.40 | 1.93 |
| Cerebrovascular diseases | | 1.10 | 0.89 | 1.37 |
| Peripheral vascular diseases | | 1.11 | 0.96 | 1.29 |
| Nephropathy | | 1.18 | 1.08 | 1.28 |
| Retinopathy | | 1.21 | 1.09 | 1.36 |
| Neuropathy | | 1.12 | 0.98 | 1.28 |
| Other obesity-related medical conditions^†^ | | 1.28 | 1.01 | 1.63 |
| Cancers (ref.: none) | |  |  |  |
|  | MS-related cancers^††^ | 1.67 | 1.28 | 2.19 |
|  | Other cancers^¶^ | 2.26 | 1.63 | 3.13 |
| All cause death (ref.: none) | | 1.92 | 1.50 |  |

Abbreviations: BMI, body mass index; MS, metabolic syndrome.

Notes:

^*^ Acute metabolic complications included diabetic ketoacidosis and hyperosmolar hyperglycemic syndrome.

^†^ Other obesity-related medical conditions included sleep apnea, bariatric surgery, and knee replacement therapy.

^††^ MS-related cancers included liver, colorectal, bladder, pancreatic, endometrial, and breast postmenopausal cancers.

^¶^ Other cancers included any malignancy such as lymphoma and leukemia, except malignant neoplasm of skin and MS-related cancers mentioned above.

^§^ The exchange rate between USD and NTD was 1:30.98 in 2023.

^||^MS-related risk factors included BMI ≥ 27 kg/m^2^, and the presence of hypertension, hyperlipidemia and diabetes.

**Supplementary Table 6. Cost multipliers for clinical characteristics of patients with metabolic syndrome (sensitivity analysis without clinical characteristics [i.e., smoking, education level, and monthly income] that had unknown data)**

| **Study variables** | | **Multiplier** | **95% CI** | |
| --- | --- | --- | --- | --- |
| Baseline annual healthcare cost (2023 USD), mean (95% CI) | | 257.44 | 190.64 | 347.65 |
| Demographics | |  |  |  |
| Age at the index date (years) (ref.: < 55) | |  |  |  |
|  | 55−64 | 1.22 | 1.09 | 1.36 |
|  | 65−74 | 1.38 | 1.22 | 1.57 |
|  | ≥ 75 | 1.41 | 1.21 | 1.65 |
| Sex (ref.: female) | | 0.99 | 0.89 | 1.10 |
| Marital and betel nut chewing status | |  |  |  |
| Marital status (ref.: single) | |  |  |  |
|  | Married | 1.04 | 0.85 | 1.27 |
|  | Divorced/widowed | 1.12 | 0.90 | 1.40 |
| Betel nut chewing status (ref.: never) | |  |  |  |
|  | Ever | 1.06 | 0.94 | 1.19 |
| Body mass index (kg/m^2^) (ref.: < 27)^\|\|^ | |  |  |  |
| 27−< 29.9 (mild obesity) | | 1.04 | 0.93 | 1.15 |
| ≥ 30 (moderate or morbid obesity) | | 1.10 | 0.98 | 1.25 |
| Comorbidity (ref.: none) | |  |  |  |
| Hypertension^\|\|^ | | 1.64 | 1.36 | 1.98 |
| Diabetes^\|\|^ | | 1.61 | 1.47 | 1.77 |
| Hyperlipidemia^\|\|^ | | 1.33 | 1.21 | 1.46 |
| Osteoarthritis | | 1.20 | 1.07 | 1.34 |
| Depression | | 2.06 | 1.41 | 3.02 |
| Complications (event-year) (ref.: none) | |  |  |  |
| Cardiovascular diseases | | 2.59 | 2.18 | 3.07 |
| Cerebrovascular diseases | | 2.07 | 1.61 | 2.67 |
| Peripheral vascular diseases | | 1.23 | 1.06 | 1.44 |
| Nephropathy | | 1.28 | 1.17 | 1.39 |
| Retinopathy | | 1.23 | 1.11 | 1.37 |
| Neuropathy | | 1.28 | 1.12 | 1.46 |
| Acute metabolic complications^*^ | | 1.92 | 1.17 | 3.15 |
| Other obesity-related medical conditions^†^ | | 2.39 | 1.84 | 3.12 |
| Cancers (ref.: none) | |  |  |  |
|  | MS-related cancers ^††^ | 2.75 | 2.05 | 3.69 |
|  | Other cancers^¶^ | 3.74 | 2.81 | 4.97 |
| Complications (state-year) (ref.: none) | |  |  |  |
| Cardiovascular diseases | | 1.63 | 1.39 | 1.92 |
| Cerebrovascular diseases | | 1.09 | 0.88 | 1.35 |
| Peripheral vascular diseases | | 1.11 | 0.97 | 1.28 |
| Nephropathy | | 1.18 | 1.08 | 1.29 |
| Retinopathy | | 1.21 | 1.09 | 1.35 |
| Neuropathy | | 1.13 | 0.99 | 1.30 |
| Other obesity-related medical conditions^†^ | | 1.28 | 1.01 | 1.62 |
| Cancers (ref.: none) | |  |  |  |
|  | MS-related cancers^††^ | 1.67 | 1.28 | 2.18 |
|  | Other cancers^¶^ | 2.27 | 1.63 | 3.16 |
| All cause death (ref.: none) | | 1.91 | 1.49 | 2.45 |

Abbreviations: BMI, body mass index; MS, metabolic syndrome.

Notes:

^*^ Acute metabolic complications included diabetic ketoacidosis and hyperosmolar hyperglycemic syndrome.

^†^ Other obesity-related medical conditions included sleep apnea, bariatric surgery, and knee replacement therapy.

^††^ MS-related cancers included liver, colorectal, bladder, pancreatic, endometrial, and breast postmenopausal cancers.

^¶^ Other cancers included any malignancy such as lymphoma and leukemia, except malignant neoplasm of skin and MS-related cancers mentioned above.

^||^MS-related risk factors included BMI ≥ 27 kg/m^2^, and the presence of hypertension, hyperlipidemia and diabetes.

**Supplementary Table 7. The Strengthening the Reporting of Observational Studies in Epidemiology (STROBE) checklist**

|  | Item No | Recommendation | Page  No |
| --- | --- | --- | --- |
| **Title and abstract** | 1 | (*a*) Indicate the study’s design with a commonly used term in the title or the abstract | 1 |
|  |  | (*b*) Provide in the abstract an informative and balanced summary of what was done and what was found | 3,4 |
| Introduction | | | |
| Background/rationale | 2 | Explain the scientific background and rationale for the investigation being reported | 5,6 |
| Objectives | 3 | State specific objectives, including any prespecified hypotheses | 6 |
| Methods | | | |
| Study design | 4 | Present key elements of study design early in the paper | 6 |
| Setting | 5 | Describe the setting, locations, and relevant dates, including periods of recruitment, exposure, follow-up, and data collection | 6-8 |
| Participants | 6 | (*a*) *Cohort study*—Give the eligibility criteria, and the sources and methods of selection of participants. Describe methods of follow-up  *Case-control study*—Give the eligibility criteria, and the sources and methods of case ascertainment and control selection. Give the rationale for the choice of cases and controls  *Cross-sectional study*—Give the eligibility criteria, and the sources and methods of selection of participants | 6-8 |
|  |  | (*b*) *Cohort study*—For matched studies, give matching criteria and number of exposed and unexposed  *Case-control study*—For matched studies, give matching criteria and the number of controls per case | NA |
| Variables | 7 | Clearly define all outcomes, exposures, predictors, potential confounders, and effect modifiers. Give diagnostic criteria, if applicable | 7-8 |
| Data sources/ measurement | 8* | For each variable of interest, give sources of data and details of methods of assessment (measurement). Describe comparability of assessment methods if there is more than one group | 6-8 |
| Bias | 9 | Describe any efforts to address potential sources of bias | 9-10, 16 |
| Study size | 10 | Explain how the study size was arrived at | NA |
| Quantitative variables | 11 | Explain how quantitative variables were handled in the analyses. If applicable, describe which groupings were chosen and why | 7-10 |
| Statistical methods | 12 | (*a*) Describe all statistical methods, including those used to control for confounding | 8-10 |
|  |  | (*b*) Describe any methods used to examine subgroups and interactions | NA |
|  |  | (*c*) Explain how missing data were addressed | 9-10 |
|  |  | (*d*) *Cohort study*—If applicable, explain how loss to follow-up was addressed  *Case-control study*—If applicable, explain how matching of cases and controls was addressed  *Cross-sectional study*—If applicable, describe analytical methods taking account of sampling strategy | 7-9 |
|  |  | (*e*) Describe any sensitivity analyses | 9-10 |

Continued on next page

| Results | | | |
| --- | --- | --- | --- |
| Participants | 13* | (a) Report numbers of individuals at each stage of study—eg numbers potentially eligible, examined for eligibility, confirmed eligible, included in the study, completing follow-up, and analysed | 10 |
|  |  | (b) Give reasons for non-participation at each stage | NA |
|  |  | (c) Consider use of a flow diagram | NA |
| Descriptive data | 14* | (a) Give characteristics of study participants (eg demographic, clinical, social) and information on exposures and potential confounders | 10,23-24 |
|  |  | (b) Indicate number of participants with missing data for each variable of interest | 23-24 |
|  |  | (c) *Cohort study*—Summarise follow-up time (eg, average and total amount) | 10 |
| Outcome data | 15* | *Cohort study*—Report numbers of outcome events or summary measures over time | 10,25 |
|  |  | *Case-control study—*Report numbers in each exposure category, or summary measures of exposure | NA |
|  |  | *Cross-sectional study—*Report numbers of outcome events or summary measures | NA |
| Main results | 16 | (*a*) Give unadjusted estimates and, if applicable, confounder-adjusted estimates and their precision (eg, 95% confidence interval). Make clear which confounders were adjusted for and why they were included | 10-12,26-29 |
|  |  | (*b*) Report category boundaries when continuous variables were categorized | 26-28 |
|  |  | (*c*) If relevant, consider translating estimates of relative risk into absolute risk for a meaningful time period | NA |
| Other analyses | 17 | Report other analyses done—eg analyses of subgroups and interactions, and sensitivity analyses | 11-12 |
| Discussion | | | |
| Key results | 18 | Summarise key results with reference to study objectives | 12 |
| Limitations | 19 | Discuss limitations of the study, taking into account sources of potential bias or imprecision. Discuss both direction and magnitude of any potential bias | 16 |
| Interpretation | 20 | Give a cautious overall interpretation of results considering objectives, limitations, multiplicity of analyses, results from similar studies, and other relevant evidence | 12-16 |
| Generalisability | 21 | Discuss the generalisability (external validity) of the study results | 12-17 |
| Other information | | | |
| Funding | 22 | Give the source of funding and the role of the funders for the present study and, if applicable, for the original study on which the present article is based | 17 |

*Give information separately for cases and controls in case-control studies and, if applicable, for exposed and unexposed groups in cohort and cross-sectional studies.

**Note:** An Explanation and Elaboration article discusses each checklist item and gives methodological background and published examples of transparent reporting. The STROBE checklist is best used in conjunction with this article (freely available on the Web sites of PLoS Medicine at http://www.plosmedicine.org/, Annals of Internal Medicine at http://www.annals.org/, and Epidemiology at http://www.epidem.com/). Information on the STROBE Initiative is available at www.strobe-statement.org.

**Supplementary Figure 1: Cumulative incidence rates of metabolic syndrome-related complications of interest**


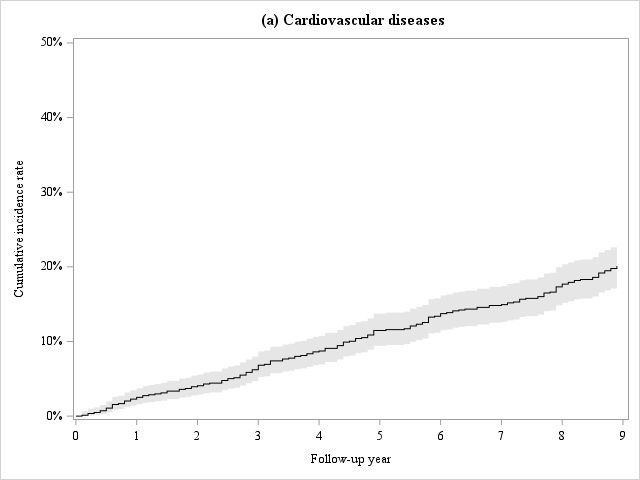


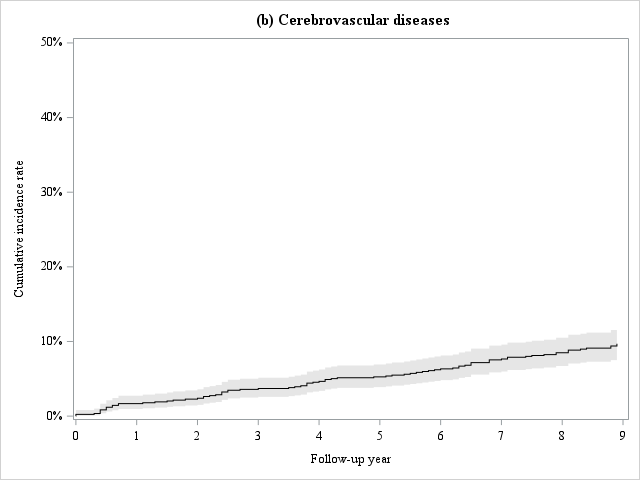

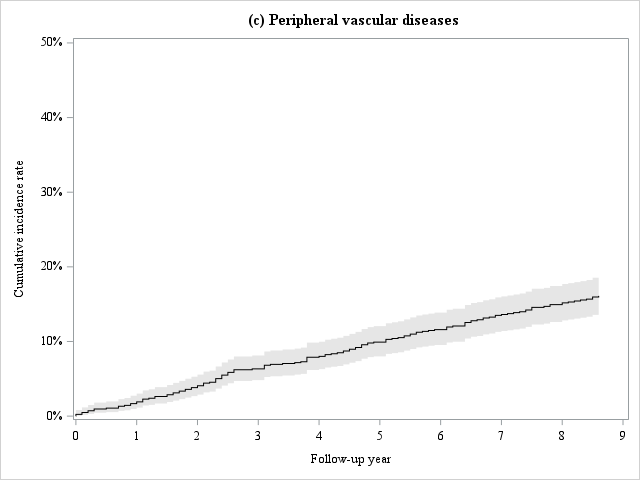

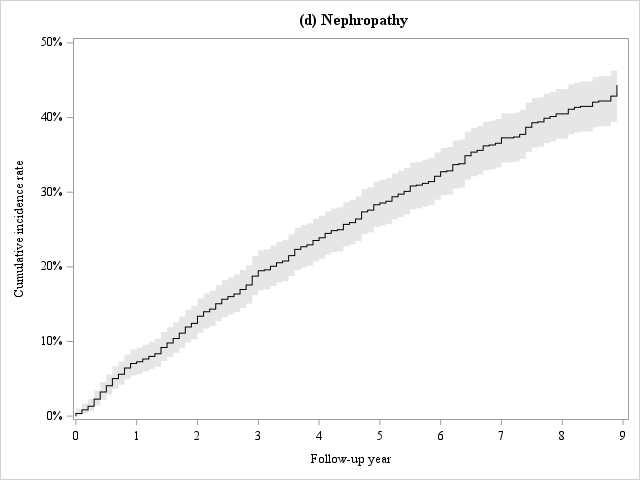

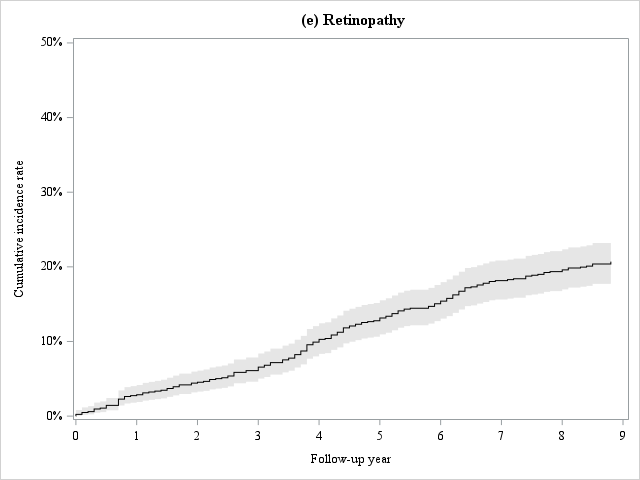

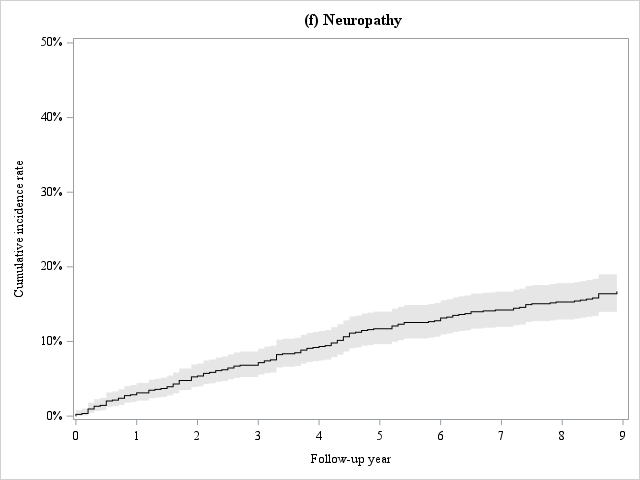

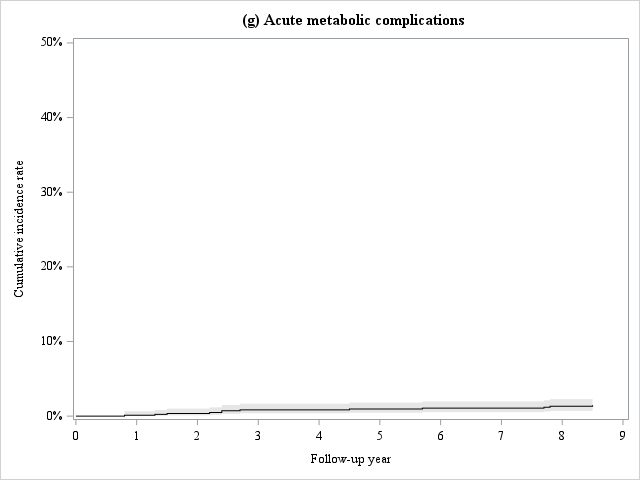

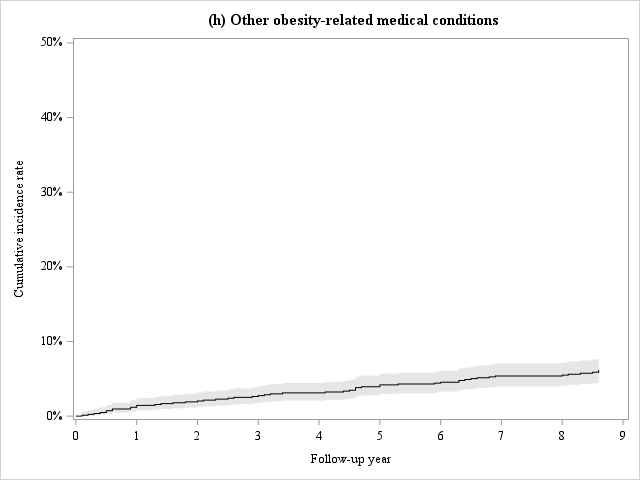

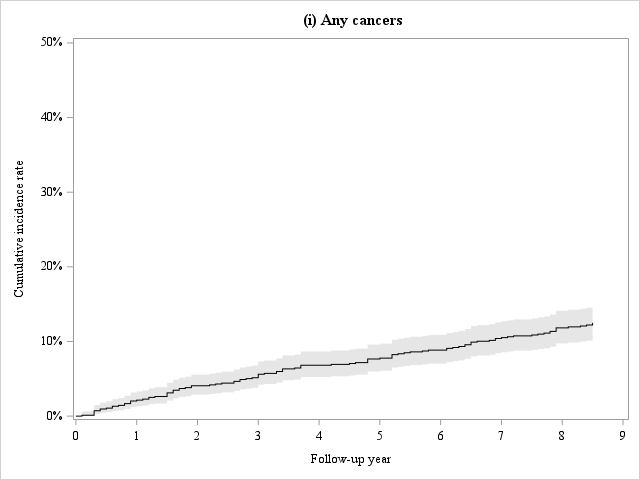

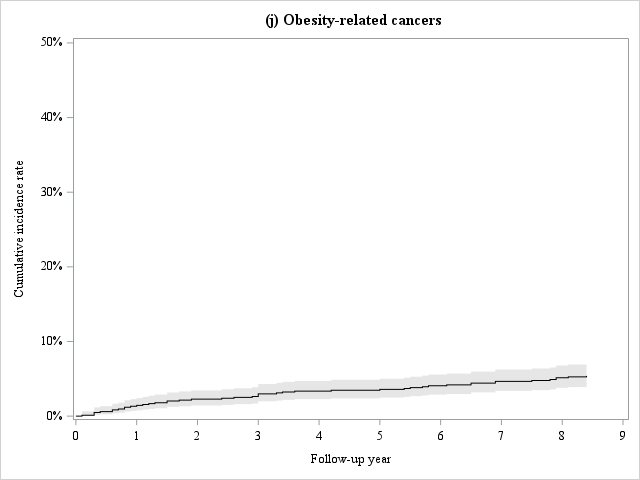

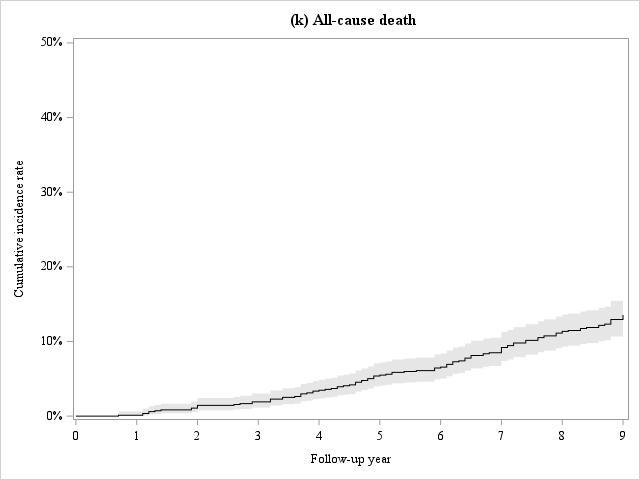


Abbreviation: MS, metabolic syndrome.

Notes:

1. The estimates (line) and 95% confidence intervals (band) of cumulative incidence rates were presented.
2. Acute metabolic complications included diabetic ketoacidosis and hyperosmolar hyperglycemic syndrome.
3. Other obesity-related medical conditions included sleep apnea, bariatric surgery, and knee replacement therapy.
4. MS-related cancers included liver, colorectal, bladder, pancreatic, endometrial, and breast postmenopausal cancers. Any cancers included MS-related and other cancers (e.g., solid tumor and lymphoma).
5. Year 0 represents the year of MS being confirmed. The cumulative incidence rates of individual complications are plotted over the years after MS was confirmed (i.e., Years 1 to 9).

**Supplementary Figure 2. Crude annual healthcare costs (per person) of (a) macrovascular complications, (b) microvascular complications, and (c) other complications, compared with those of non-complications (“NC”) from baseline year (before complication occurred; Year 0) up to 6 years of follow-up (Years 1−6)**


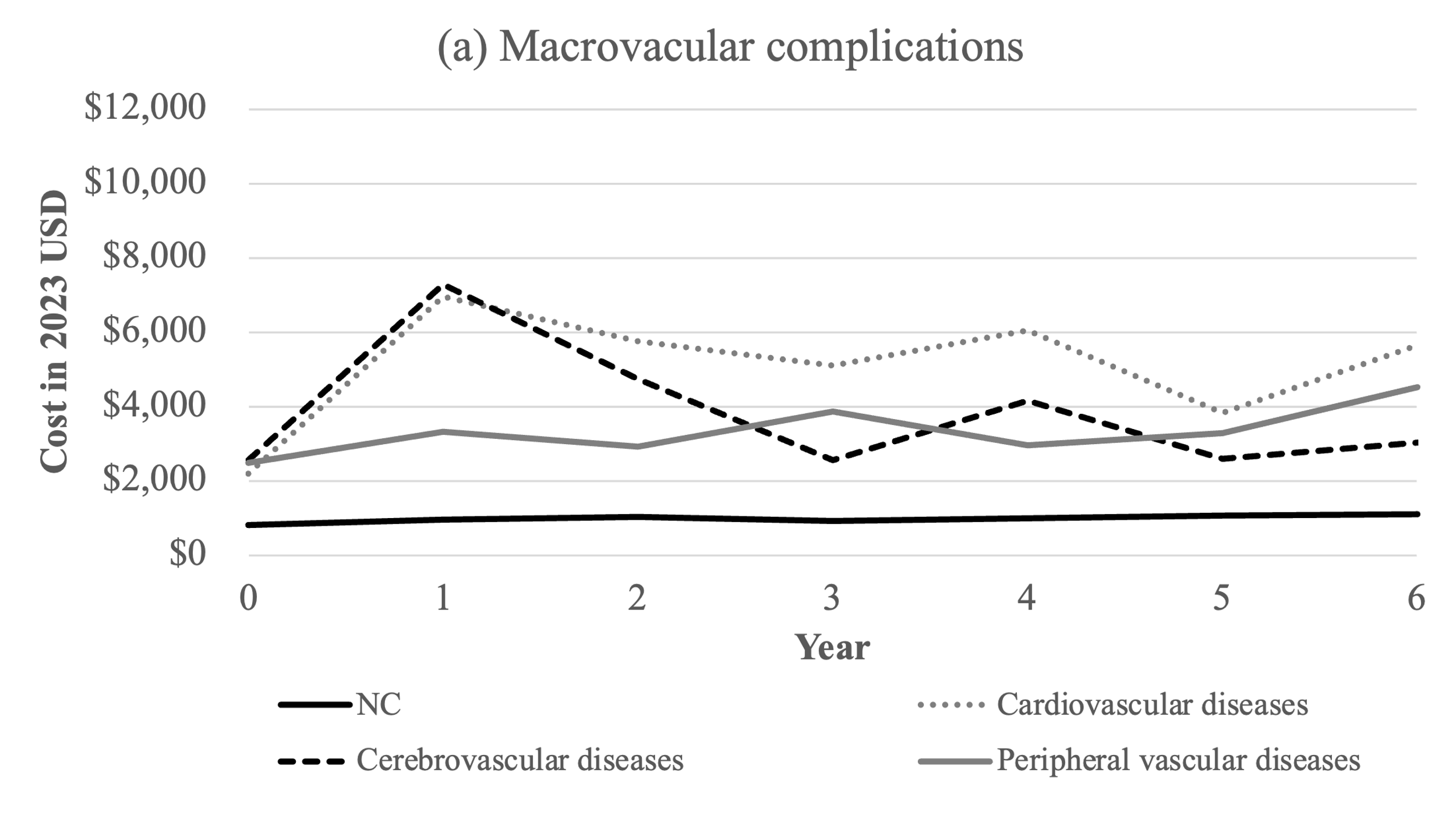


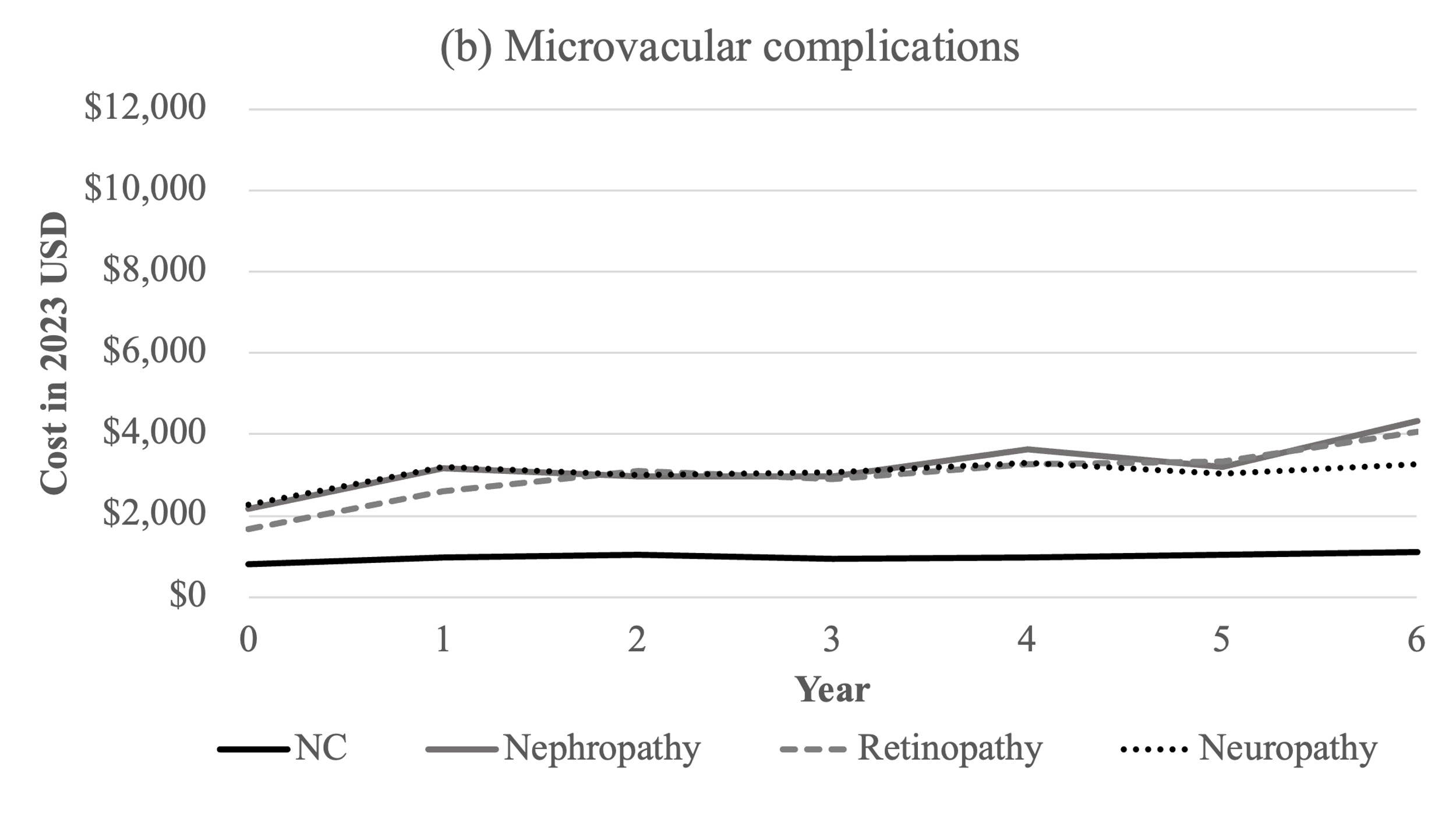


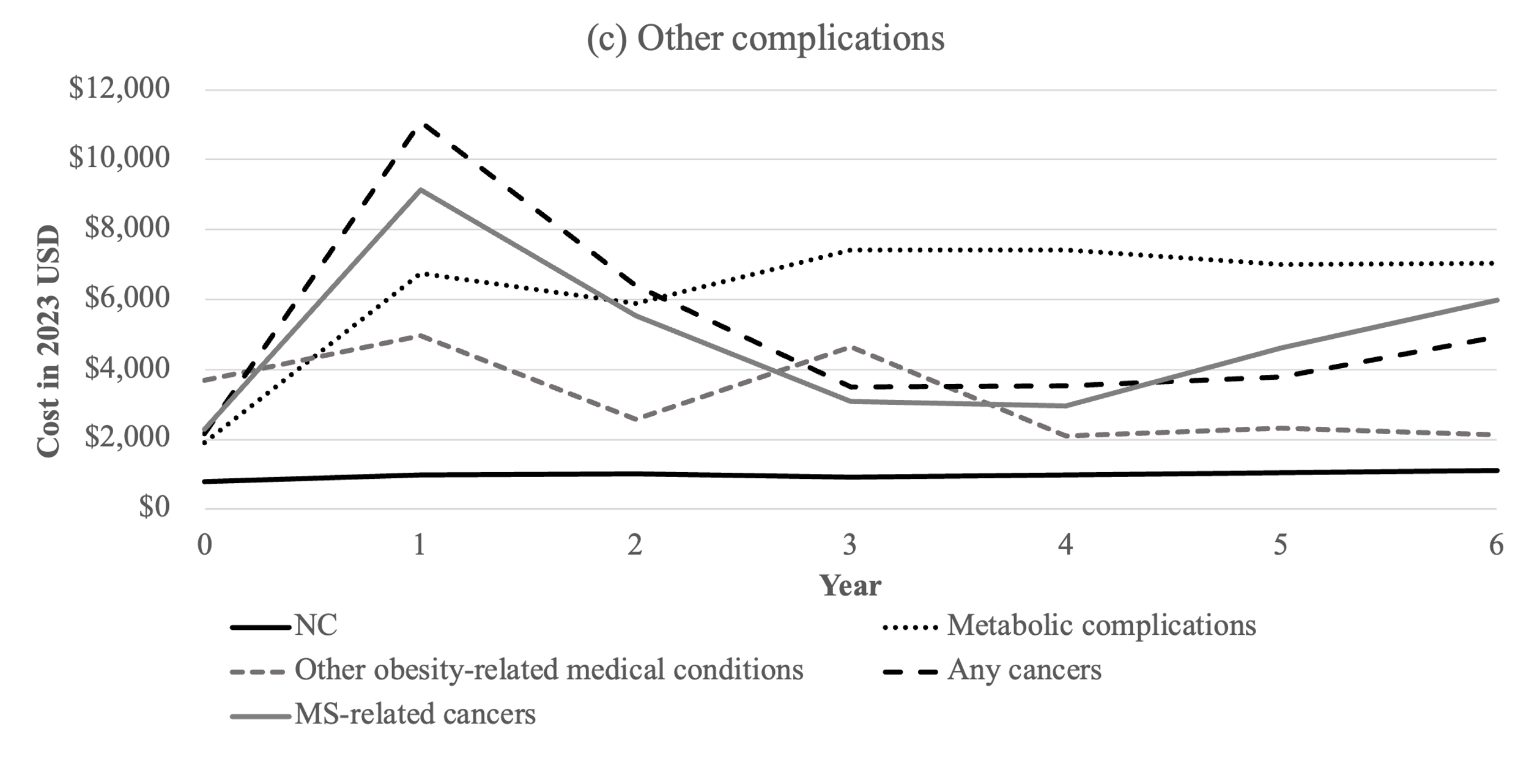


Abbreviations: NC, no complications; MS, metabolic syndrome.

Notes:

1. Acute metabolic complications included diabetic ketoacidosis and hyperosmolar hyperglycemic syndrome.
2. Other obesity-related medical conditions included sleep apnea, bariatric surgery, and knee replacement therapy.
3. MS-related cancers included liver, colorectal, bladder, pancreatic, endometrial, and breast postmenopausal cancers. Any cancers included MS-related and other cancers (e.g., solid tumor and lymphoma).
4. Year 0 refers to the year before the complication of interest occurred. Year 1 is the year when the complication occurred (i.e., event-year) and Years 2−6 are the years after the complication occurrence (i.e., state-years).
5. Due to small sample size (e.g., less than 10% of study participants for individual complications) in the 7th year of follow-up, the costs were measured over only 6 years of follow-up.
